# Diagnosis of the mechanisms of different types of discordances between phylogenies inferred from nuclear and mitochondrial markers

**DOI:** 10.18699/VJ20.634

**Published:** 2020-07

**Authors:** A.A. Poroshina, D.Y. Sherbakov, T.E. Peretolchina

**Affiliations:** Limnological Institute of Siberian Branch of the Russian Academy of Sciences, Irkutsk, Russia; Limnological Institute of Siberian Branch of the Russian Academy of Sciences, Irkutsk, Russia; Limnological Institute of Siberian Branch of the Russian Academy of Sciences, Irkutsk, Russia

**Keywords:** mitochondrial introgression, incomplete lineage sorting, ancient lakes, sympatric speciation, parapatric speciation, disagreements between phylogenies, Lake Baikal, митохондриальная интрогрессия, неполная сортировка родословной, древние озера, симпатрическое видообразование, парапатрическое видообразование, разногласия между филогениями, озеро Байкал

## Abstract

In ancient freshwater lakes, an abnormally large species diversity is observed. The mechanisms that generated extremely high biodiversity in the ancient lakes have not been sufficiently studied and remain only partially known. Sequences of environmental changes in highly complex ecosystems such as Lake Baikal, may induce sophisticated combinations of microevolutionary processes. These processes are likely to result in unusual “patterns” of genetic variability of species. The most unusual patterns include the ones when speciation is followed by incomplete lineage sorting as well as mitochondrial or nuclear introgression. All these phenomena are diagnosed by comparing the topologies of phylogenetic trees inferred from molecular markers of evolution located in mitochondria and nuclei. Mitochondrial and nuclear introgression is a particularly interesting and complex case, which is the process of incorporating the gene alleles of one species into the gene pool of a sister species due to interspecific hybridization (introgressive hybridization). In many cases, existing methods for molecular phylogenetic analysis do not automatically allow the observed patterns of polymorphism to be explained and, therefore, cannot provide hypotheses that would explain the mechanisms which resulted to these patterns. Here we use adaptive dynamics models to study neutral molecular evolution under various scenarios of interaction between sister species and the environment. We propose and justify a set of criteria for detecting how two evolutionary trees may differ, with a special focus on comparing a tree inferred from nuclear DNA to one from mitochondrial DNA. The criteria react to branching pattern and branch lengths, including relative distances from ancestral lineages. Simulations show that the criteria allow fast and automated detection of various types of introgression, secondary breaches of reproductive barriers, and incomplete lineage sorting.

## Abstract (ru)

В древних пресноводных озерах наблюдается аномально большой видовой состав организмов. Механизмы, которые обусловили формирование биоразнообразия в древних озерах, еще недостаточно изучены. Микроэволюционные процессы, приводящие в конечном итоге к видообразованию, включают в себя в качестве элементарных процессов изменения численности особей, миграцию и репродуктивную изоляцию. В природных условиях, особенно в такой сложной экосистеме, как экосистема Байкала, комбинации микро- эволюционных процессов могут носить самый причудливый характер и порождать необычные «узоры» ге- нетической изменчивости видов. К наиболее необычным следам сложных видообразовательных процессов относятся унаследованный полиморфизм, а также митохондриальная и ядерная интрогрессии. Эти явления диагностируются на основании сравнения топологий филогенетических деревьев, построенных по ядерным и митохондриальным молекулярным маркерам эволюции. Особенно интересный и сложный случай – мито- хондриальная и ядерная интрогрессия, представляющая собой процесс включения аллелей генов одного вида в генофонд сестринского вида при межвидовой гибридизации (интрогрессивная гибридизация). Часто существующие методы анализа генетического полиморфизма не позволяют автоматически находить объясне- ние наблюдаемых картин полиморфизма и, следовательно, предлагать гипотезы, которые бы раскрыли механизмы, породившие эти картины. В настоящей работе мы используем модели адаптивной динамики для изу- чения нейтральной молекулярной эволюции при различных сценариях взаимодействия между сестринскими видами и окружающей средой. Мы предлагаем набор критериев для определения того, как могут различаться два эволюционных дерева, построенных с использованием последовательностей ядерной и митохондриаль- ной ДНК. Моделирование показывает, что критерии позволяют быстро и автоматически выявлять различные типы интрогрессии, вторичные нарушения репродуктивных барьеров и неполное расхождение видов.

## Introduction

Significant advances in gathering the data on genetic polymorphisms of all kinds of organisms have shed light on important features of the evolutionary process. For example, recent technique, allow one to study previously cryptic features of the mechanisms, generating and maintaining the diversity of life. Among the most challenging foci of modern evolutionary studies are the hyper-diverse and geographically constrained ancient freshwater lakes (Lake Baikal in East Siberia, Lake Tanganyika in East Africa etc). These lakes are inhabited by rapidly evolving species assemblages generated by adaptive radiation, mostly in sympatry, and ultimately responding to the fast and powerful environmental challenges generated by global changes (Brooks, 1950; Sherbakov, 1999; Salzburger et al., 2014). Studies of speciation processes in ancient lakes revealed numerous cases of presumably complicated evolutionary histories and therefore many unexpected patterns of genetic diversity. The most striking are the cases of dramatic discordance between the patterns resulting the studies of mitochondrial and nuclear DNA described in (Nevado et al., 2009; Sturmbauer et al., 2010; Kéver et al., 2018).

Although these phenomena are well and long known to exist in many species, their explanations involve the assumption of large-scale range shifts (Toews, Brelsford, 2012; Schön, Martens, 2012) and thus are hardly applicable to the situations of sympatric or parapatric speciation responsible for the most of the species diversification in relatively small and closed ecosystems. A systematic study of disagreements between mitochondrial and nuclear phylogenies requires a formal procedure automating search for such cases, such as testing significance of the disagreement and modelling of evolutionary scenarios likely to cause generation of the discrepancies between nuclear and cytoplasmic phylogenies. Here we describe fast simultaneous analysis of two tree topologies allowing one to detect a discordance, test its significance and diagnose its type. We test this approach on a set of trees resulting simulations of evolutionary events and on the real-world data set on endemic to Lake Baikal gastropods of genus Baicalia.

## Materials and methods

**Individual based modeling.** Individual-based models (Grimm, Railsback, 2005) simulating evolution of two sister species of diploid organisms possessing both mitochondrial and nuclear DNA markers were designed as described in (Semovski et al., 2004). Differential responses of the sister species to the same environmental challenges were modelled by pre-setting independent curves of environmental niche capacity for the two sympatrically occurring species. Model also allows independent variation of gene flows between the species thus mimicking asymmetric or symmetric breach of the reproductive barrier during defined periods of time. Each simulation was succeeded by collection of certain amount (usually 100 of each to make it comparable to an experimental study) of marker sequences from the same “individuals”, saved in separate files and maximum likelihood trees were inferred with PhyML version 3.3.20180129 (Guindon et al., 2010), using the model of molecular evolution set to JC because of the simulation settings. Tree comparisons were performed with custom Python scripts using ete2 library (Huerta-Cepas et al., 2010).

**Types of discordances between mitochondrial and nuclear markers.** Discordance between nuclear and mitochondrial trees should be declared when their branching patterns differ significantly so that one of them looks distorted strongly if compared to the other. We test following kinds of phylogeny discordances of two sister groups of organisms may occur:

- splitting into two species;
- introgression is when on the one tree all groups form separate clades, while on the other tree one group appears inside the cluster formed by the other group. In other words, the common for one group allele becomes fully substituted with the allele originated from the other group;
- inherited polymorphism (incomplete lineage sorting) is when due to resent breach of reproductive barrier both groups acquire alleles from the other ones. Discriminating between different types of branching order discordances.

**Fig. 1.**
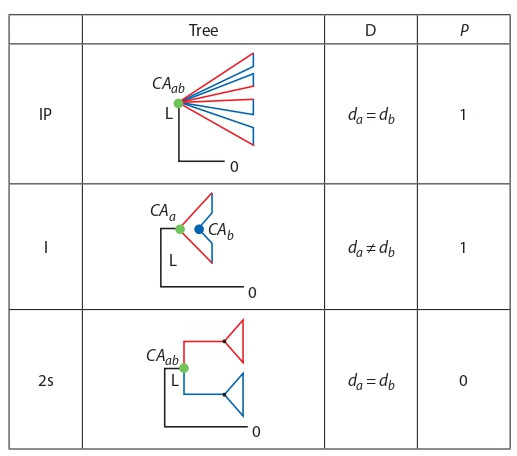
Schematic representation of the distortions to the tree topology resulting the inherited polymorphism (incomplete lineage sorting) and full introgression as compared to the unspoiled separation of two sister species.

In order to detect the cases of introgressions while comparing data sets, we had developed the test, which was used then to optimize the processing of fairly large amounts of data. The test employs three values estimated from the phylogenies (Fig. 1). The first value is the corrected mutational distance between the common ancestors of the two species (nodes LA and LB) and the most basal node CA (common ancestor of the two species). In the case of inherited polymorphism all three points in fact are the same point and thus all three distances are equal to zero. Therefore if all three distances insignificantly differ from zero one may assume incomplete sorting of ancestral lineages. Obviously all three distances would have significant lengths if unaccomplished divergence of the two species took place. We can also find the distance between CA and L (d ) for two species and find their difference (D). With inherited polymorphism and separation into two types, the distances da and db will be approximately equal.

The second value useful for detecting of introgression is the sum of all distances from all representatives of the same species to the most recent common ancestral node of the tree (EΣ if the number of specimens of the two species analyzed is very far from equal, the average distance E by a species must be used). In spite of any accomplishments this value should be approximately equal if molecular clock hypothesis holds. The latter condition may be tested separately. The significantly different from zero value of the difference between EA and Eb determined for one molecular marker, i. e. mitochondrial gene while remaining approximately equal for other marker(s) points at full introgression (see Fig. 1).

The possibility of a dramatic violations of molecular clock hypothesis as well as numerous other reasons make it necessary to support the hypothesis on the introgression independently. Here we propose to use for that purpose the test for monophyly of the clades in question. We employed the simplest form of the test as it is implemented in ete2 and ete3 libraries for Python (http://etetoolkit.org/). Based on the topology of the tree it calculates the coefficient of monophyly, P. It becomes 0 in case of a monophyletic clade and 1 in case of polyphyly.

The values and designations involved in the analysis are: P – inherited polymorphism; I – introgression; 2s – two species; P – coefficient of polyphyleticity (0: monophyletic, 1: polyphyletic); CA – closest common ancestor; L – furthest descendant of the common ancestor indexed by the species; d – distance (along the tree) from CA to L indexed by the species.

It is important to note that the protocol (pipeline) described here makes it easy to test directly the statistical significance of the diagnosis. This may be done by applying it to the bootstrap replicates of the original data set followed by odds ratio test to estimate the support to the conclusion.

In the case of the full introgression all sequences of one type appear as the ingroup(s) to the recipient type. The other marker yields monophyletic clusters consisting of a single type of sequences. The four cases above may be diagnosed by measuring the distances between the common ancestors of sequences as they appear on the tree and comparing them to distances from the common ancestors to the common root in combination with the monophyleticity test for the both sister groups. Indeed, the inner branch between the common ancestors of two groups passes the node defined by the outgroup in all cases except for complete introgression. In the latter case the common ancestor of the recipient group is connected to the outgroup via the common ancestor of the donor group. In this case the donor group is always polyphyletic, while the recipient may be both monophyletic or polyphyletic if the introgression occurred more then, once or multiple donor’s lineages were involved. Mutual introgression from the recent bi-directional hybridization generates the latter pattern in both directions. In this case each type of sequences appears in the other cluster as an ingroup.

Introgression differs from incomplete lineage sorting because in former case common ancestors of the invader (donated) lineages would be significantly younger then the common ancestor of the recipient. In contrast, incomplete sorting of ancestral lineages results in the absence of separate clusters for the two groups in case of one of the markers. The simplest cause of the latter may be the insufficient rate of molecular evolution of the marker affected.

In all cases the internal branching order must be robustly supported, and the branches crucial for the diagnosis must have significant lengths. Therefore testing the diagnosis significance simply turns into testing the significance of the sum of branch lengths between common ancestors of the groups (species) and the most ancestral node of the tree. There are several methods of estimating the confidence limits of the branch length (Felsenstein, Felenstein, 2004; Anisimova, Gascuel, 2006).

Additional feature helping to diagnose the distortions of tree topology is the monophyleticity of the groups. In the case of the lack of any discordance, the mutual correspondence must hold between the groups and clades on the tree.

**Fig. 2.**
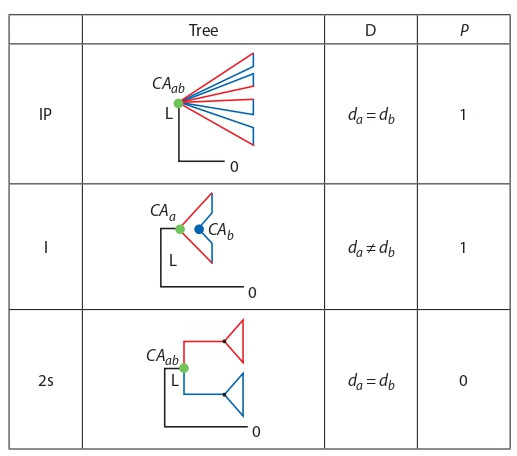
The respective position of the most recent common ancestors (MRCA) in trees inferred from nuclear and mitochondrial introgressions. Upperraw (a): trees inferred from nuclear markers, lower raw (b): trees inferred from mitochondrial markers.

If the tree is rooted by outgroup (Fig. 2, scheme of a fully resolved phylogeny of two groups showing designations used in this paper) CAA and CAB labelled with colored dots designate the common ancestors of the respective groups, O is their common ancestor as it is defined by the outgroup) linked to the common ancestor of species A and B with their common ancestors designated as it is shown on the same figure, the relative position of the three nodes will distinguish between full introgressions if taken together with the results of testing the groups for monophyleticity. All possible outcomes are summarized in Table 1 (L is the distance between the common ancestor of a groups to the common ancestor of the two groups).

**Table 1.**
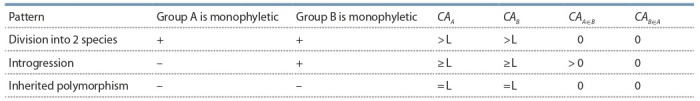
Diagnostic of the tree distortions (nuclear or mitochondrial)

**Baicalia sequences.** Sampling locations of Baicalia specimens are specified in (Peretolchina et al., 2007). Genomic DNA was extracted from muscle tissue using a modified method described earlier (Sokolov, 2000). Gene fragments of mitochondrial cytochrome c oxidase subunit 1 (COI) were amplified using primers L1490 (5ʹ-GGTCAACAAATCATAA AGATATTGG-3ʹ) and H2198 (5ʹ-TAAACTTCAGGGTGAC CAAAAAATCA-3ʹ) (Folmer et al., 1994); fragments of the internal transcribed spacer (ITS1) were amplified using primers Kp-2F (5ʹ-AAAAAGCTTCCGTAGGTGAACCTGCG- 3ʹ) and 5.8SR (5ʹ-AGCTTGGTGCGTTCTTCATCGA-3ʹ) (Nazar, Roy, 1978). An average of 1–3 μL of DNA extracted was amplified in a 25 μL reaction using BioMaster HS-Taq PCR Kit (Biolabmix, Russia) under the conditions recommended by the manufacturer. The conditions for amplification, electrophoresis and amplicon purification are those in T.E. Peretolchina et al. (2007). GenBank accession numbers of ITS1 sequences: FJ598711, FJ598712, FJ598715–FJ598723, FJ598727–FJ598732, FJ598735–FJ598741, FJ598743– FJ598745, FJ598748–FJ598760, FJ598762–FJ598771, FJ598832–FJ598848; of COI sequences: Z92995 (Zubakov et al., 1997), HQ113269–HQ113278, DQ436384–DQ436443, GU22640–GU22649, KT885116, FJ749133.

**Software availability.** All custom software used in this work is available from https://github.com/dysh/MRDR.

## Results and discussion

**Fig. 3.**
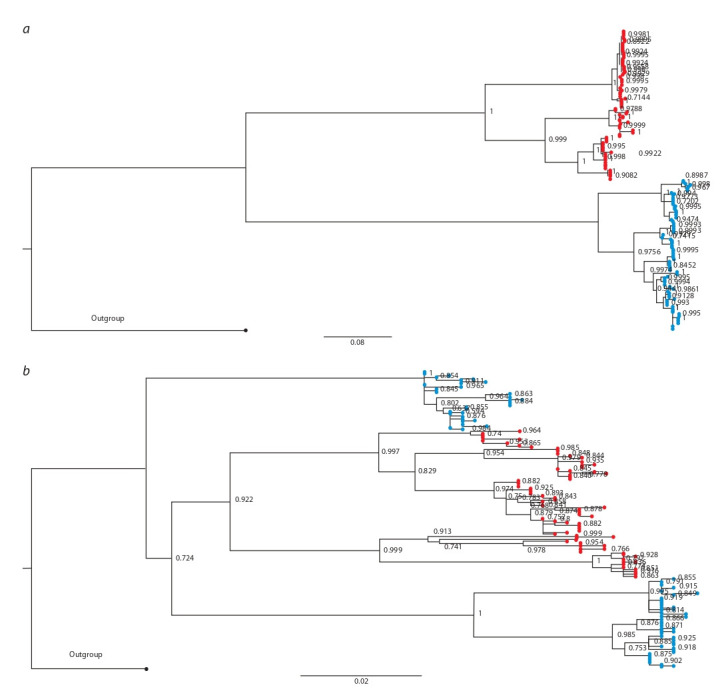
Phylogenetic trees inferred from simulated data sets. a – the tree inferred from “sequences” evolving as neutral nuclear marker; b – the tree based on “sequences” evolved according to the rules specific for mitochondrial DNA. The aLRT supports are given in the nods of trees.

We have used several example phylogenies resulted from computer experiments where various scenarios were used to generate different patterns of disagreements between maternally inherited mitochondrial and nuclear DNA of diploid organisms. The simulations involved differential responses of sister species to the changes of environmental capacity accompanied by periods of reproductive isolation breaches. Each simulation produced two sets of “DNA sequences” (Semovski et al., 2004) used for tree inferences subsequently compared to each other using the procedure described here. All kinds of disagreements between the trees were obtained in course of these simulations and successfully classified using the distances between ancestral roots and tests for motophyleticity (Table 2). An example of full mitochondrial introgression is shown on Fig. 3 (circles designate common ancestors. The trees are rooted with the starting sequence used in the simulation).

**Table 2.**
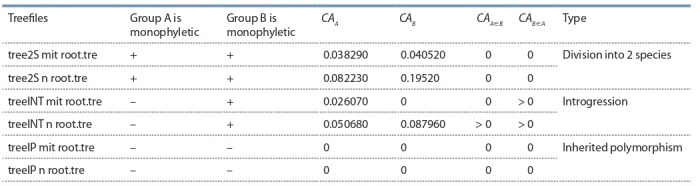
Metrics of the ML trees inferred from simulated data

**Fig. 4.**
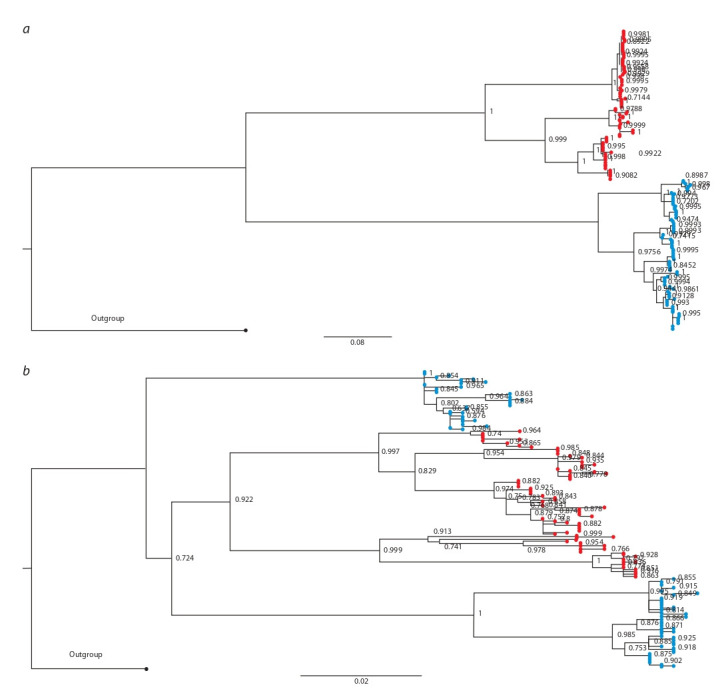
Phylogenetic trees inferred from DNA sequences of a mitochondrial marker COI (a) and nuclear marker ITS1 (b) for three sister species of gastropods belonging to genus Baicalia (B. carinata (blue), and B. dybowskiana (red)). The trees are rooted by outgroups consisting of all paralogous sequences belonging to other Baicaliinae. The aLRT supports are given in the nods of trees.

**Experimental data example: genus Baicalia.** Gastropod genus Baicalia belonging to the subfamily Baicaliinae endemic to Lake Baikal consists of five species diverged from the common ancestor relatively recently in confines of the lake (Sherbakov, 1999). Species of this genus differ morphologically and ecologically. The most remarkable ecological difference between them is their substrate – dependent mating behavior. Both nuclear and mitochondrial markers have been used in phylogenetic inferences involving the species of Baicalia (Zubakov et al., 1997; Peretolchina et al., 2007; Sitnikova et al., 2016). Dramatic discrepancies between phylogenies inferred from several nuclear markers and mitochondrial markers are reported to be typical for the whole group and for Baicalia in particular (Peretolchina et al., 2007; Sitnikova et al., 2016). Here we have reproduced phylogenetic inferences using the data set consisting of two markers of different inheritance mode belonging to three Baicalia species: B. carinata, B. dybowskiana and B. turriformis from (Peretolchina et al., 2007). Two separate phylogenies have been obtained for the nuclear gene ITS1 and mitochondrial COI. Maximum likelihood trees obtained differed from each other (Fig. 4).

The two species, B. dybowskiana and B. turriformis, appear as monophyletic separate clades in a phylogeny inferred from nuclear sequences. According to the tree inferred from the sequences of mitochondrial origin both B. dybowskiana and B. turriformis become in-groups to B. carinata. However, their clusters are separated from each other. This example illustrates that discrepancies between phylogenies may be relative and complicated if traced in more than two groups: while there are clear indications of full mitochondrial introgressions from B. carinata to B. turriformis and to B. dybowskiana, if the representatives of latter two species are subjected to phylogenetic inference in the absence of B. carinata there is no discrepancy between the trees inferred from different markers. Interestingly, the two introgressions were not simultaneous and occurred with a large time gap.

## Conclusion

We present here a simple and fast procedure allowing one to distinguish automatically between contrasting patterns of disagreement between trees inferred for the same groups of organisms using different sets of data. In this communication we concentrate on the comparison between trees inferred from DNA sequences of mitochondrial and nuclear origin. First, we define different kinds of disagreements between the tree topologies (introgression, ancestral polymorphism) and propose the set of criteria, which may be estimated from a phylogeny. The approach is based on the estimating distances between common ancestors of groups defined externally, for example, by species identity of their members. Two trees are used as the input, the groups are tested if they are monophyletic and then set of distances between clusters is measured. At this point it is possible to test for the statistical significance of the distortions and their diagnoses using any appropriate approach such as various kinds of bootstrapping.

This procedure is required for any modeling efforts aiming at the elucidation of ecological circumstances favoring different types of disagreements between trees inferred for the same organisms. It is interesting to note, that the same procedure is potentially applicable to the cases when several sets of loci of the same mode of inheritance give rise to dramatically different trees due to selection or adaptation-guided acquisition from sister taxa. The discordance detection procedure proposed here is fast and sufficient to browse transcriptomes in search of sequence cliques evolving accordingly to each other but differently from other large sets of sequences.

## Conflict of interest

The authors declare that the research was conducted in the absence of any commercial or financial relationships that could be construed as a potential conflict of interest.
